# Functional Changes of Therapeutic Antibodies upon Exposure to Pro-Oxidative Agents

**DOI:** 10.3390/antib11010011

**Published:** 2022-02-02

**Authors:** Maxime Lecerf, Robin Lacombe, Alexia Kanyavuz, Jordan D. Dimitrov

**Affiliations:** Centre de Recherche des Cordeliers, INSERM, CNRS, Sorbonne Université, Université de Paris, 75006 Paris, France; maxime.lecerf@inserm.fr (M.L.); robinlacombe99@gmail.com (R.L.); alexia-tavares@orange.fr (A.K.)

**Keywords:** therapeutic antibodies, antibody developability, oxidation, antibody polyreactivity, ferrous ions, hypochlorite

## Abstract

Therapeutic monoclonal antibodies have exerted a transformative impact on clinical practice in last two decades. However, development of a therapeutic antibody remains a complex process. Various physiochemical and functional liabilities can compromise the production or the therapeutic efficacy of antibodies. One of these liabilities is the susceptibility to oxidation. In the present study, we portrayed an oxidation-dependent vulnerability of immunoglobulins that can be of concern for therapeutic antibodies. By using a library of 119 monoclonal IgG1 molecules, containing variable domain matching clinical-stage antibodies, we demonstrated that a substantial number of these molecules acquired antigen-binding polyreactivity upon exposure to ferrous ions. Statistical analyses revealed that the potential for induction of polyreactivity by the redox-active metal ions correlated with a higher number of somatic mutations in V genes encoding variable domains of heavy and light immunoglobulin chains. Moreover, the sensitive antibodies used with biased frequencies particular V gene families encoding variable domains of their light chains. Besides the exposure to ferrous ions the induction of polyreactivity of therapeutic antibodies occurred after contact with an unrelated pro-oxidative substance—hypochlorite ions. Our data also revealed that induction of polyreactivity by pro-oxidative agents did not impact the binding of antibodies to their cognate antigens. The results from this study may contribute for better selection of antibody therapeutics with suitable developability profiles.

## 1. Introduction

Heterogeneity of sequence and various post-translational modifications of the variable (V) immunoglobulin domains endows antibodies (Abs) with the potential to recognize a vast diversity of antigens [1,2]. This heterogeneity also endows different Ab molecules with idiosyncratic physicochemical properties such as distinct thermodynamic stability, tendency for aggregation, capacity for binding to numerous unrelated antigens (polyreactivity), etc. Not surprisingly, recognition of a single epitope can be achieved by Abs with different sequence of V domains and hence with distinct physicochemical characteristics. These considerations are especially important regarding development of monoclonal therapeutic Abs. It is well known that identification of an Ab molecule that bind with high affinity to a given target of therapeutic interest is largely not sufficient for successful entry into the clinical practice [3]. Since the process of development of therapeutic Abs is complex and resource-consuming [4], the presence of any unfavourable physicochemical properties of Abs can compromise the endeavour at different levels. Various liabilities, or developability issues, of therapeutic Abs have been identified [3]. These issues can affect the recombinant protein production (low expression yields), Ab storage (tendency for aggregation and low stability) or pharmacokinetics and pharmacodynamics of Abs that are administered in patients (binding polyreactivity) [3,5,6,7]. Presence of different types of liabilities of monoclonal Abs encouraged development of plethora of computational and analytical techniques for assessment of drug candidates and early detection of the problematic characteristics [3,6,8,9,10,11,12,13].

An important liability of therapeutic Abs molecules is the susceptibility to oxidation [3,14]. This vulnerability can affect Abs both during their production as well as after administration in patients (as for example after exposure to pro-oxidative molecules at sites of inflammation). The oxidative modifications of Ab molecule can lead to appearance of other liabilities, e.g., increase in immunogenicity, decrease in thermodynamic stability and increase in tendency for aggregation [3].

Importantly, it was demonstrated that immune repertoires of healthy humans have a fraction of Abs that acquire potential to bind with high affinity to multiple unrelated antigens (polyreactivity) after exposure to different pro-oxidative agents [15,16,17,18,19]. These redox-agents include some biologically relevant substances such as heme, iron ions, hypochlorite, etc. [15,16,18,20,21]. Since Ab polyreactivity is considered an important developability issue for Abs that may compromise the pharmacokinetics and increase the incidence of secondary effects [22], the emergence of this Ab attribute in some therapeutic molecules following exposure to pro-oxidative conditions might be of concern. Notably, these Abs would not manifest antigen binding polyreactivity in their native state and hence might not be detected by the developability assays designed for evaluation of polyreactivity.

The frequency of Abs that acquire polyreactivity upon exposure to iron ions among clinical-stage therapeutic Abs is unknown. It is also not known what the molecular features of V domains are that pre-determine the potential of certain Abs to change their binding behaviour and interact with many unrelated target antigens after exposure to redox-active metal ions. To address these questions, we assayed a repertoire of 119 monoclonal therapeutic Abs that are currently approved for clinical use or are in clinical trials for acquisition of binding polyreactivity after exposure to ferrous ions. These analyses revealed that a considerable number of the therapeutic Abs acquire polyreactivity upon contact with the pro-oxidative metal ions. The sequence analyses uncover numerous traits of the V regions that correlate with sensitivity to ferrous ions. This study provides an important information of relevance for therapeutic Ab development and optimization.

## 2. Materials and Methods

### 2.1. Recombinant Abs

In the present study, we used 117 samples with variable region sequences corresponding to Abs that are approved for clinical use or are currently under investigation in Phase II and Phase III clinical trials (kindly provided by Adimab LLC, Lebanon, NH, USA). To this repertoire we added 2 IgG1 Ab preparations for clinical use of Rituximab and Trastuzumab (F. Hoffmann-La Roche AG, Basel, Switzerland). Detailed information about the used repertoire of recombinant Abs was provided in the article of Jain et al. [6]. All recombinant Abs were expressed as human IgG1 subclass.

### 2.2. ELISA—Screening of Repertoire of Therapeutic Abs for Induction of Polyreactivity by Ferrous Ions

The experiment was performed as previously described in [23] 96-well polystyrene plates NUNC MaxiSorp™ (Thermo Fisher Scientific) were coated for 1 h at room temperature with human recombinant factor VIII (Advate, Baxter), human plasma-derived C3 (CompTech, Tyler, TX, USA) and recombinant LysM polypeptide of AtlA from *Enterococcus faecalis* (kindly provided by Dr. Stephan Mesnage, University of Sheffield, Sheffield, UK). The coating was performed with protein diluted to 3 μg/mL in PBS. Next, the plates were blocked by incubation for 1 h at room temperature with solution of 0.25% Tween 20 in PBS. After blocking the plates were incubated with therapeutic Abs. To this end, each Ab was first diluted in PBS to 100 μg/mL and treated with final concentration of 200 μM FeSO_4_ (Sigma-Aldrich, St. Louis, MO, USA, before use—preparation of 10 mM stock in deionized water) for 10 min and then Abs were diluted to 20 μg/mL in PBS-T and incubated with the immobilized proteins for 90 min at room temperature. As an internal control each plate was incubated with 250 μg/mL of native pooled human IgG (Endobuline Baxter USA). For assessment of binding of each recombinant Ab to the studied proteins, the plates were first washed 5 times with PBS-T and then incubated for 1 h at room temperature with horseradish peroxidase-conjugated anti-human IgG (a mouse monoclonal Ab clone JDC-10, Fc-specific, Southern Biotech, Birmingham, AL, USA), diluted 3000× in PBS-T. Following washing with PBS-T, the immunoreactivity of the Abs was revealed by addition of peroxidase substrate, *o*-phenylenediamine dihydrochloride (Sigma-Adrich). The measurement of the optical density at 492 nm was conducted with a microplate reader (Infinite 200 Pro, Tecan, Männedorf, Switzerland) after stopping of the enzyme reaction by the addition of 2 M HCl.

The reactivity of 113 native Abs to the same set of proteins was published in a previous study [23]. As the reactivity of all ferrous ion-exposed 119 native Abs was assessed simultaneously and in same conditions, we used here the previously published data obtained with 113 native Abs combined with 6 additional native Abs as a reference.

### 2.3. Statistical Analyses

Amino acid sequences of variable regions of mAbs used in this work were retrieved from sequence data in the study of Jain et al. [6]. The CDRs and framework regions of heavy and light chains V domains of Abs were determined with IMGT/V-QUEST (http://www.imgt.org (accessed on 18 November 2021)) by using human germ-line genes as a reference. The numbers of amino acids substitutions, insertions and deletions between Abs sequence and inferred germ-line gene were extracted from the IMGT/V-QUEST result and referred as mutations.

The reactivity of each Ab to each studied protein (Factor VIII, C3 and LysM) was defined as the ratio between the absorbance obtained for the ferrous ion-treated Ab versus the absorbance obtained for the native Ab. These ratios were subjected to non-parametric Spearman correlation analysis using GraphPad Prism v.9 software (La Jolla, CA, USA). Ferrous ion-induced polyreactivity was defined as the mean of the ratios of increased reactivity for the three tested Ag.

Spearman correlation *ρ* values were presented as heat maps along with corresponding *p* values in order to identify correlations between mean Fe^2+^ ion-induced polyreactivity of Abs and (i) the number of mutations in variable regions, (ii) the number of individual amino acid resides or their sums grouped by types (polar, acidic, basic, aromatic and hydrophobic) in each CDR or framework region, (iii) grand average of hydropathy (GRAVY) value in each CDR or framework region (data computed using GRAVY Calculator www.gray-calculator.de (accessed on 10 November 2021)) and (iv) the isoelectric point (pI) value in each CDR or FR (data computed using the “IPC2.peptide.Conv2D” parameter of the Isoelectric Point Calculator 2.0 http://ipc2.mimuw.edu.pl (accessed on 10 November 2021 )).

### 2.4. ELISA—Interaction of Rituximab and Trastuzumab with Panel of Antigens

Ninety six-well polystyrene plates NUNC MaxiSorp™ (Thermo Fisher Scientific) were coated for 90 min at room temperature with human recombinant factor VIII (Advate, Baxter), human plasma-derived C3 (CompTech), porcine thyroglobulin (Sigma-Aldrich), bovine histone 3 (Sigma-Aldrich), recombinant LysM polypeptide of AtlA from *Enterococcus faecalis* and LPS from *E. coli* O55:B5 (Sigma-Aldrich). The coating was performed with protein diluted to 2 μg/mL (Factor VIII and C3) or 10 μg/mL (thyroglobulin, histone 3, LysM and LPS) in PBS. Afterwards, the plates were blocked by incubation for 1 h at room temperature with a solution of 0.25% Tween 20 in PBS. Rituximab and Trastuzumab (both stock solutions in PBS) were first diluted to 1 mg/mL (6.7 μM) in PBS and left intact or exposed for 10 min at room temperature to 500 μM FeSO_4_ or to 200 μM NaOCl (Sigma-Aldrich). After native substances and those treated with pro-oxidative Abs were serially diluted from 3.35 to 0.003 μM and incubated for 1 h at room temperature. The following steps of the ELISA are identical with those described above.

### 2.5. Immunoblot Analysis

Total lysate of *Bacillus anthracis* at 1 mg/mL (kindly provided by Dr. Stephan Mesnage, University of Sheffield, UK) was loaded (100 μL per gel) on a 4–12% gradient NuPAGE Novex SDS-PAGE gel (Thermo Fisher Scientific). Following electrophoretic separation, proteins were transferred on nitrocellulose membranes (iBlot gel transfer stacks, Thermo Fisher Scientific) by using electrotransfer system (iBlot, Invitrogen, Thermo Fisher Scientific). After the membranes were blocked overnight at 4 °C in PBS containing Tween 0.1% (PBS-T). For treatment of the therapeutic Abs, Rituximab and Trastuzumab were diluted to 1 mg/mL (6.7 μM) in PBS and exposed to 500 μM FeSO_4_ or 200 μM NaOCl for 10 min at room temperature. Native and treated Abs were then diluted to 100, 33.3 and 11.1 μg/mL in PBS-T and incubated with the immobilized bacterial proteins using the Miniblot channel system (Immunetics, Cambridge, MA, USA). The incubation with Abs was performed for 2 h at room temperature. Nitrocellulose membranes were washed (6 × 10 min) with excess of PBS-T before incubation for 1 h at room temperature with an alkaline phosphatase-conjugated anti-human IgG (polyclonal goat IgG, Southern Biotech, Birmingham, AL), diluted 3000× in PBS-T. Membranes were then extensively washed (6 × 10 min) with PBS-T and the immunoreactivity revealed by the using SigmaFAST^TM^ NBT/BCIP substrate system (Sigma-Aldrich).

### 2.6. Size-Exclusion Chromatography

Stocks of Rituximab and Trastuzumab (from therapeutic preparations dialyzed against PBS) were diluted at 1 mg/mL in PBS and subject—or not—to ferrous treatment (500 µM FeSO_4_ final concentration) or to NaOCl treatment (200 µM NaOCl final concentration). After 30 min incubation on ice, 100 µL of native and treated Abs (not dialysed) were injected on a Superose 6 Increase column (Cytiva, Marlborough, MA, USA) mounted on an Äkta Purifier 10 (Cytiva) and previously equilibrated with at least 3 column volumes of PBS. Elution was carried out with PBS at 0.5 mL/min and monitored at 280 nm. Raw data were exported from Unicorn software (Cytiva) and elution profiles were overlaid using GraphPad Prism v.9 software.

### 2.7. Flow Cytometry

Daudi and Raji—human B-cell lymphoma lines were cultured in RPMI supplemented with GlutaMax (ThermoFisher Scientific, Walthman, MA, USA) and 10% foetal calf serum (FCS) and incubated at 37 °C 5% CO_2_. Cells were washed in cold RPMI without FCS and dispensed at 300,000 cells per well, in 90 µL per well of a round-bottom 96-well plate. Rituximab was diluted at 1 mg/mL in PBS and subjected—or not—to ferrous ions treatment (500 µM FeSO_4_ final concentration) or to NaOCl treatment (200 µM NaOCl final concentration). After 30 min of incubation, the cells were incubated with PBS only, or with 10 µg/mL of native Rituximab, ferrous ion-treated or of hypochlorite ion-treated Rituximab. After 30 min of incubation on ice, cells were washed with 100 µL of cold PBS and resuspended in 100 µL of goat anti-human IgG Fc–PE (ThermoFisher Scientific, Walthman, MA, USA) diluted 500× in cold PBS. After 30 min of incubation on ice, cells were washed with 100 µL of cold PBS and resuspended in 200 µL PBS. Flow cytometry analysis was performed on an LSRII (BD, Franklin Lakes, NJ, USA) with the FSC, SSC and blue 562-588-A parameters. The population of interest was selected by plotting FSC-A and SSC-A. Homogenous population of interest was gated and doublets were eliminated. Fluorescence intensities of 10,000 events were recorded in the blue 562-588-A channel Data analysis was made with FlowJo v10.8 (BD, Franklin Lakes, NJ, USA).

### 2.8. Surface Plasmon Resonance Analyses

Surface plasmon resonance (SPR)-based assay (Biacore 2000, Cytiva Biacore, Uppsala, Sweden) was used to assess the interaction of Trastuzumab to its target Her2. First, recombinant Her2 (ErbB 2, Abcam, Cambridge, UK) was covalently immobilized on the surface of the CM5 sensor chip (Biacore, Cytiva) using standard amine coupling protocol. Thus, the protein was diluted in 5 mM maleic acid solution, pH 4 to 10 μg/mL and injected over the sensor surface pre-activated by a mixture of EDC/NHS (from Amine-coupling kit, Biacore Cytiva). Unconjugated groups were blocked by injection of 1 M ethanolamine HCl for 4 min. The final immobilization density of Her2 was approximately 800 RU.

All analyses were performed with HBS-EP (10 mM HEPES pH 7.2; 150 mM NaCl; 3 mM EDTA, and 0.005% Tween 20). The flow rate of the buffer during interaction analyses was set at 30 μL/min. Trastuzumab at 1 mg/mL was treated with 500 μM FeSO_4_ or to 200 μM NaOCl for 10 min at room temperature. After two-fold serial dilutions of native and oxidant-treated Trastuzumab in HBS-EP starting from 10 to 0.039 nM were conducted. These concentrations of Ab were injected over immobilized Her2. The association phase of the binding of Ab was monitored for 4 min; the dissociation phase was monitored for 5 min. The regeneration of the sensor surface after each Ab infection was performed by 4 M solution of MgCl_2_ contact time 30 s. All binding analyses were performed at temperature of 25 °C. The binding of native or treated Trastuzumab to control sensor surface (containing only carboxymethylated dextran) was subtracted from the binding during data processing. The assessment of binding kinetics was performed by BIAevaluation version 4.1.1 Software (Biacore) by using the Langmuir binding model.

## 3. Results

### 3.1. Reactivity towards Unrelated Proteins of 119 Therapeutic Abs before and after Exposure to Fe^2+^ Ions

Previous studies demonstrated that the exposure of pooled human IgG to ferrous ions results in considerable functional changes, i.e., gain in reactivity towards numerous unrelated human and bacterial proteins [16,17,21]. As the modification of antigen reactivity and the presence of polyreactivity are undesirable for therapeutic Abs, we analysed a repertoire of 119 clinical-stage Abs for sensitivity to pro-oxidative substance—Fe^2+^ ions. All studied Abs were expressed as IgG1. They have identical heavy constant chains and differ only by the sequence of their V domains. Most of Abs in the repertoire (107/119) have a κ-light chain. Among the studied Abs, 47 have been approved for use in clinics and 72 are presently in phase II or III clinical trials. The reactivity of Abs to human proteins—Factor VIII and C3 and a protein from *Enterococcus faecalis*—LysM, was elucidated by ELISA. In another study, we investigated the reactivity towards the same set of proteins of 113 therapeutic Abs from the same repertoire before and after exposure to heme [23]. Since the binding of heme- and ferrous ion-treated therapeutic as well as all 119 native Abs were assessed simultaneously, in the present study we used previously published values of reactivity of native Abs to Factor VIII, C3 and LysM [23] to evaluate the gain in reactivity upon exposure to ferrous ions.

**Figure 1:**
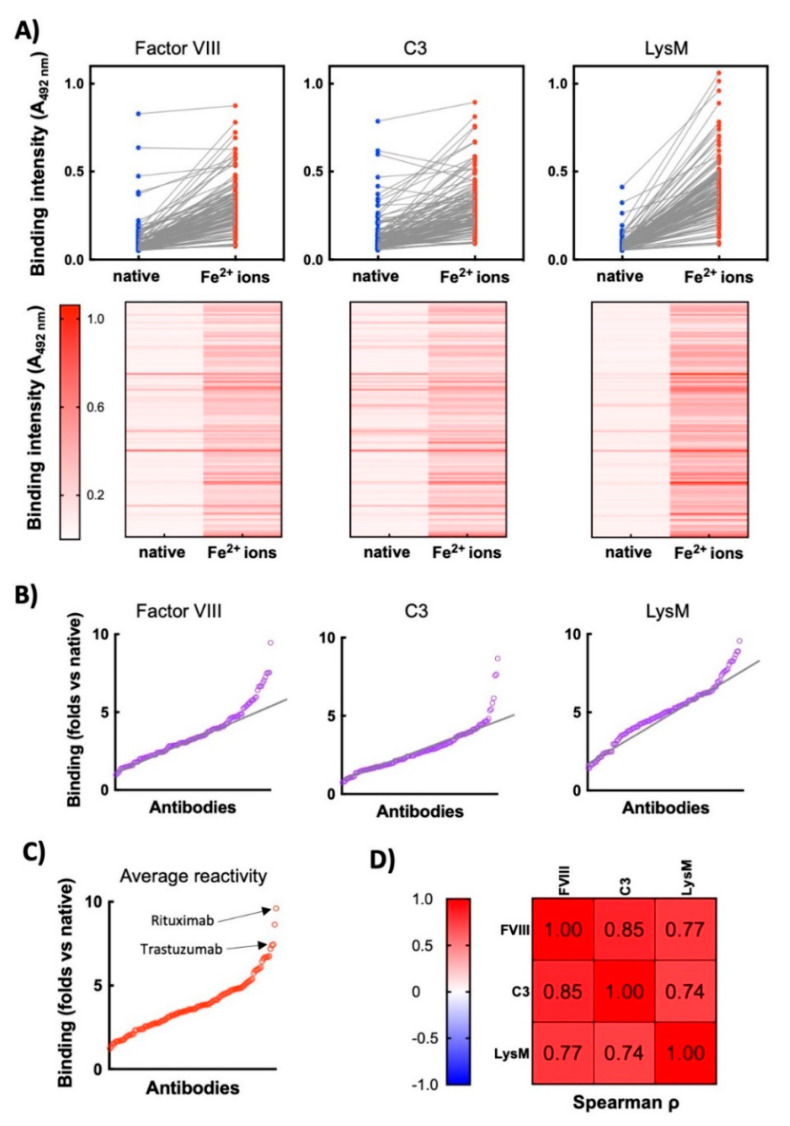
Exposure of therapeutic Abs to ferrous ions results in induction of antigen-binding polyreactivity. (**A**) ELISA analyses of reactivity of 119 monoclonal therapeutic IgG1 Abs to immobilized proteins—human Factor VIII, human C3, and LysM from *E. faecalis. Upper panels*—the blue circles represent the reactivity of individual Abs in their native state; the red circles depict reactivity of Abs after exposure to ferrous ions. Each circle indicates the average binding intensity of a single Ab to a given antigen obtained from duplicate measurements. The reactivity of 113 of native Abs is taken from previous study [23]. *Lower panels*—heat maps showing the intensity of binding of each therapeutic Ab (in native form and after exposure to Fe^2+^ ions) to the indicated proteins. Reactivity of each Ab in the repertoire is represented as a horizontal line. Higher intensity of red indicates more substantial binding of Ab. (**B**) Fold increase in antigen-binding intensity after exposure to ferrous ions. The graphs show the ratio of reactivity of each Ab after exposure to Fe^2+^ ions versus reactivity of Ab in the native state towards Factor VIII, C3 and LysM. Each circle signifies one Ab. The graphs are generated from data presented in 1A. The black lines indicate the approximate trend in augmentation in the binding reactivity of repertoire towards indicated antigens. (**C**) Average increase in reactivity after exposure of therapeutic Abs to ferrous ions. The graph presents average values of fold increase binding of each Ab to the three unrelated antigens after exposure to ferrous ions. (**D**) Correlation analyses of Ab reactivities to different antigens. The table presents the correlation coefficients obtained after Spearman correlation analyses of binding reactivities of Fe^2+^-ion-treated Abs towards different antigens. All correlations are significant with *p* < 0.001.

Thus, the binding of each monoclonal Ab in its native form and after exposure to Fe^2+^ ions was compared. As can be observed on Figure 1, for many of the Abs the treatment with ferrous ions resulted in no or a negligible change in binding to the studied proteins. However, the data revealed that the repertoire contains a fraction of Abs that acquired a considerable binding potential to the proteins only after exposure to pro-oxidative metal ions. Certain Abs demonstrated binding to Factor VIII and C3 in their native forms (Figure 1A), an observation that can be explained by their natural polyreactivity. By plotting the values of fold increase in protein binding (ratio of reactivity of a given Ab after exposure to Fe^2+^ ions versus reactivity of the same Ab in its native state), it was obvious that the repertoire presented a gradual increase in the sensitivity to the pro-oxidative agent (Figure 1B) A group of Abs, however was characterized with prominent binding to the studied targets—identified by displaying reactivity beyond the gradual linear augmentation of the binding intensity (Figure 1B). Similar trend was observed when the averaged gain in reactivity of each Ab towards the three different antigens was plotted (Figure 1C). The most sensitive therapeutic Abs to ferrous ions (in descending order of gain of reactivity) were: Rituximab; Visilizumab; Vedolizumab; Trastuzumab; Inotuzumab; Imgatuzumab; Palivizumab; Motavizumab; Parsatuzumab, and Dinutuximab. Next, we compared whether the gain of the reactivity of Abs to a given protein correlates with a gain of reactivity to other unrelated proteins (Figure 1D). A strong correlation in Fe^2+^ ion-induced reactivities towards the three studied proteins was observed, suggesting the redox-active metal ions induce polyreactivity of vulnerable Abs. Collectively these data indicate that in a set of clinical-stage Abs there is a fraction of molecules that acquire polyreactivity following exposure to redox-active metal ions.

### 3.2. Analyses of the Sequence Characteristics of the V Region of Therapeutic Abs Contributing for Sensitivity to Ferrous Ions

To obtain a better understanding of the effect of pro-oxidative agent on the reactivity of Abs, we investigated sequence characteristics of V domains of heavy and light immunoglobulin chains (V_H_ and V_L_). A nonparametric *Spearman* analysis was applied to assess the correlation between the average intensity of Fe^2+^ ion-induced reactivity of Abs to three unrelated proteins with the number of somatic mutations or the frequency of particular amino acid residues in CDR and framework regions of V domains. In addition, correlation analyses of Fe^2+^ ion-induced binding intensity of Abs with overall hydrophobicity (GRAVY index) and theoretical isoelectric point of V domains was performed.

**Figure 2:**
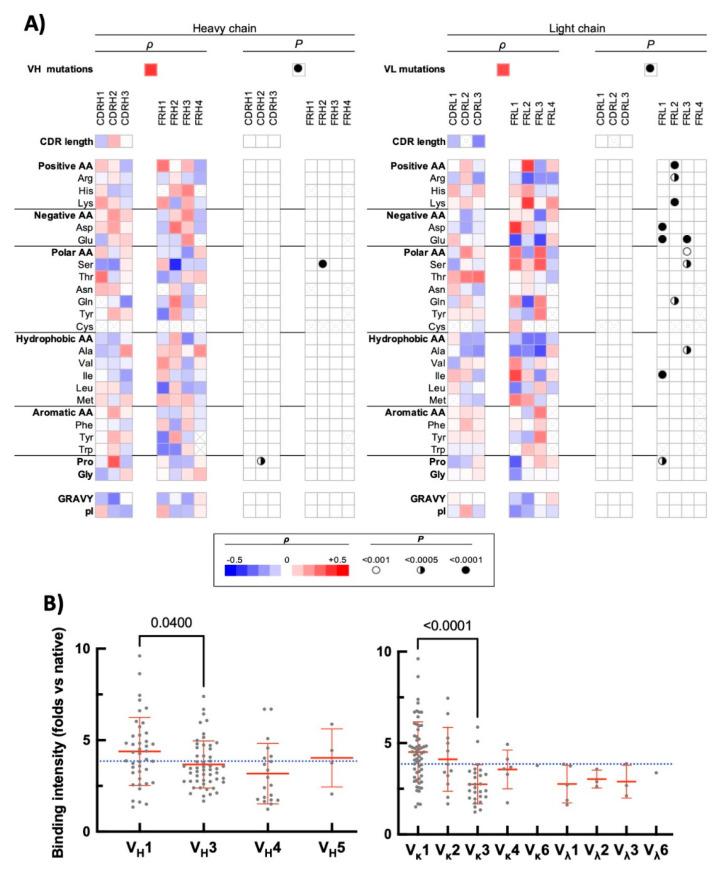
Correlation of sequence features of the variable domains of therapeutic Abs with induced polyreactivity by ferrous ions. (**A**) Heat maps depict the results from Spearman correlation analyses of the average intensity of increase in the binding of the therapeutic Abs to unrelated proteins after exposure to Fe^2+^ ions (shown on Figure 1C) with the sequence characteristics of V_H_ (**left** panels) and V_L_ (**right** panels). Following sequence characteristics were used in correlation analyses: number of somatic mutations in V genes; number of particular amino acids, or types of amino acid residues in CDR and framework regions; overall hydrophobicity (GRAVY index) and values of isoelectric points of V domains (pI). The red colour in the heat maps indicates the positive value of the correlation coefficient (ρ), the blue—negative. The intensity of colours signifies the difference of ρ from 0. The summary of statistical significance (*p* values) is shown on the right of each heat map. The symbol “×” indicates that the corresponding amino acid residue is absent in the studied sequence. (**B**) Frequency distribution of usage of V genes encoding heavy (**left** panel) and light (**right** panel) V domains as a function of average fold increase in the antigen-binding intensity of Abs gained following exposure to ferrous ions. Antigen reactivity of each Ab is presented as a single grey circle. Rituximab and Trastuzumab have VH and VL genes corresponding to mouse IGHV1-12*01 and IGLV1-40*01 as well as human IGHV3-66*01 and IGKV1-39*01, respectively. In our study, we represented the genes of the chimeric Ab Rituximab as the closest human analogues, i.e., IGHV1-46*01 and IGKV1-39*01. Statistical analyses were performed by using Mann–Whitney nonparametric test.

The analysed repertoire consists of 50 Abs that have entirely human V regions. Sixty-two Abs are humanized, six are chimeric, and one has murine V regions. We assessed the number of mutations in the V domains using inferred human germ-line genes as references (determined through IMGT database https://www.imgt.org/ (accessed on 18 November 2021)). This permitted evaluation of how the degree of divergence from the human germ-line sequence (intrinsic or due to Ab engineering) could affect the sensitivity to pro-oxidative metal ions. The Spearman analyses revealed a significant (cut-off value of *p* < 0.001) positive correlation between the number of mutations in V_H_ domain and the intensity of binding to unrelated antigens after exposure of Abs to ferrous ions (ρ = 0.39, *p* < 0.0001, Figure 2A). Similarly, the sensitivity of the therapeutic Abs to the metal ions positively correlated with a higher number of somatic mutations in V_L_ domain (ρ = 0.35, *p* < 0.0001, Figure 2A). In addition, the correlation analyses revealed that sensitivity to ferrous ions of the therapeutic Abs was significantly associated only with few sequence traits in V_H_ domain (Figure 2A): there was a positive correlation with number of Pro residues in CDR H2 (ρ = 0.32, *p* < 0.0005, average usage of Pro in CDR H2 by entire Ab repertoire: 0.46 ± 0.5) and a negative correlation with number of Ser residues in framework region 2 (ρ = −0.41, *p* < 0.0001, average usage of Ser in FR2 by entire Ab repertoire: 0.55 ± 0.72). Interestingly, sensitivity of the therapeutic Abs to ferrous ions correlated with far larger number of sequence features of V_L_ domain (Figure 2A). It is noteworthy that all significant correlations were found to affect the frequency of amino acid residues that are situated in the framework regions of V_L_. This result strongly suggests that the potential of Abs to acquire polyreactivity upon exposure to Fe^2+^ ions is mediated mainly by more conservative parts of the V_L_ domain. These regions in most of the cases are not directly involved in antigen recognition. Since the variation of sequence of the framework region could reflect usage of different V gene families encoding the V_L_ domain, we next analysed the frequency distribution of gene usage. As can be observed on Figure 2B, there was less pronounced differences in frequency of usage of V genes encoding the V_H_ domain in relation to sensitivity to ferrous ions (the two most frequent gene families, i.e., V_H_1 and V_H_2 were compared by the Mann–Whitney test and significant difference was detected *p* = 0.04). Abs that demonstrated high reactivity to studied proteins after exposure to Fe^2+^ ions utilized with the significantly higher frequency V_H_1 gene family (Figure 2B). Notably, the difference in the usage of the V genes encoding the V_L_ domain were considerably more pronounced as compared with the V_H_ domain. Thus, Abs that use gene family Vκ1 were characterized with higher sensitivity to the effect of ferrous ions (Figure 2B). On the other hand, Abs that used Vκ3 had lower than average induced polyreactivity after exposure to ferrous ions (Figure 2B). Taken together, these data imply that Abs that are using a particular V gene family for V_L_ and have higher number of somatic mutations in both V_H_ and V_L_ are more vulnerable to the effect of pro-oxidative metal ions. At this stage, however, it is impossible to apprehend how a particular sequence trait or mutation load endow Abs with higher sensitivity to ferrous ions.

### 3.3. Correlation Analyses between Heme and Ferrous Ion-Induced Polyreactivity of Therapeutic Abs

**Figure 3:**
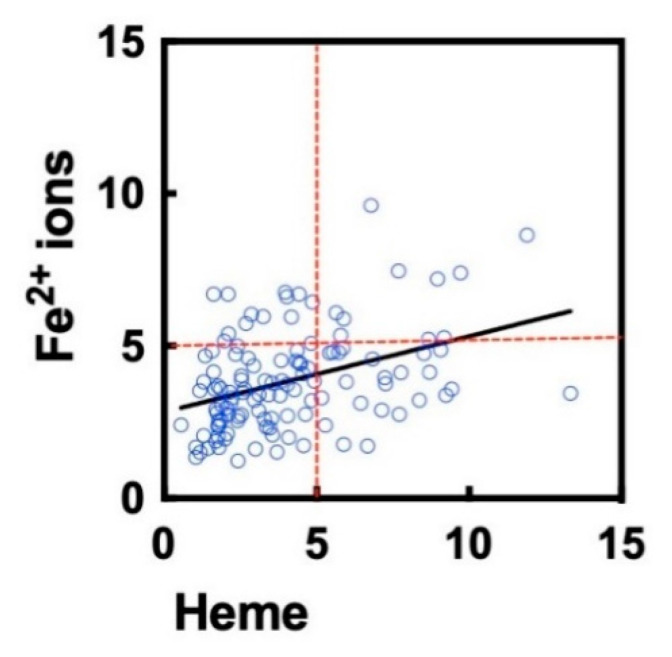
Correlation of heme- and ferrous ion-induced polyreactivity of clinical-stage Abs. The graph depicts the correlation (Spearman analyses) of the fold increase in the antigen-binding intensity (averaged gain in reactivity for three unrelated proteins) after treatment of Abs with heme or ferrous ions. The reactivity of each Ab is presented as an individual circle. The correlation was characterized by Spearman coefficient ρ = 0.38 and *p* < 0.0001. The data for heme-treated Abs were taken from a previously published study [23].

In a previous study we demonstrated that the repertoire of clinical-stage therapeutic Abs contain a fraction that acquire polyreactivity after exposure to iron-containing cofactor molecule, heme [23]. To assess whether the identical set of Ab molecules are influenced by both ferrous ions and heme, we correlated the average binding intensity of treated Abs. A significant positive correlation (ρ = 0.38, *p* < 0.0001) between the effect of the two substances was observed (Figure 3). The correlation analyses implied, however, that there are Ab molecules in the repertoire that show preferential sensitivity for heme or for Fe^2+^ ions.

### 3.4. Analyses of the Interaction of Individual Therapeutic Abs with Unrelated Antigens following Exposure to Pro-Oxidative Agents

**Figure 4:**
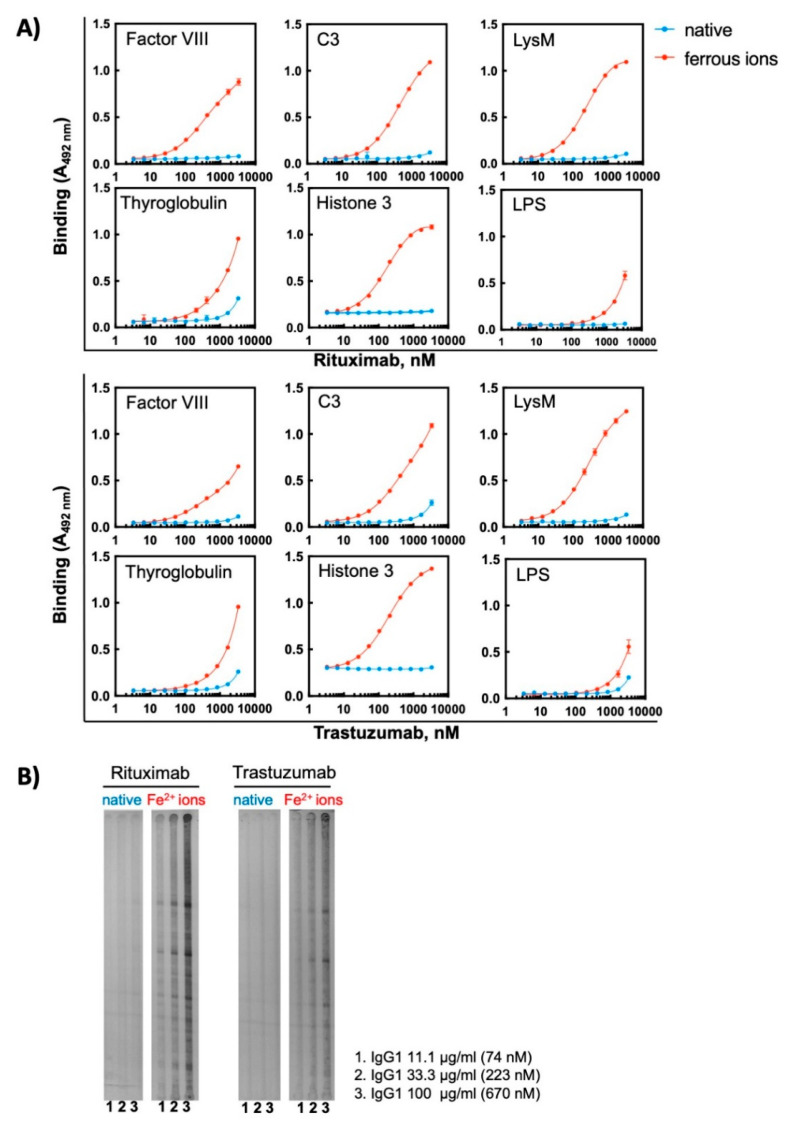
Treatment of therapeutic Abs—Rituximab and Trastuzumab—with ferrous ions results in induction of antigen-binding polyreactivity. (**A**) ELISA assay was used to evaluate the reactivity of native and Fe^2+^ ion-exposed Rituximab or Trastuzumab. The Abs at 1 mg/mL (6.7 μM) were treated with 500 μM final concentration of FeSO_4_. After native and iron ion-treated Abs were serially diluted 3350—3.27 nM and incubated with the indicated proteins and LPS from *E. coli* immobilized on ELISA plates. Each symbol represents the averaged binding intensity obtained from triplicate samples ± SD. (**B**) Immunoblot analyses of reactivity of native and ferrous ion-exposed Rituximab and Trastuzumab to antigens from total lysate from *Bacillus anthracis*. Abs were treated at 1 mg/mL (6.7 μM) with 500 μM FeSO_4_. Next native and treated Abs were diluted to 100, 33 and 11 μg/mL and incubated with the immobilized antigens using the *Immunetics* miniblot system.

To further elucidate the effect of oxidizing agents on therapeutic Abs, we selected for additional analyses two monoclonal Abs—Rituximab and Trastuzumab—that exhibited a high sensitivity to ferrous ions and acquired pronounced binding polyreactivity. First, we analysed the reactivity of these Abs to the extended panel of antigens. The binding of different concentrations of each Ab in its native state and following exposure to Fe^2+^ ions was compared by ELISA. In their native forms the therapeutic Abs demonstrated absence or only negligible binding to studied model antigens (Figure 4A). The exposure of these therapeutic Abs to Fe^2+^ ions, however, resulted in profound impact on the reactivity. They acquired binding potential for all studied protein antigens. The reactivity to non-proteinic antigen, i.e., LPS from *E. coli* was weakly affected by the pro-oxidative metal ions (Figure 4A). The induction of polyreactivity of Abs was confirmed by Western blot analyses, where the binding of different concentrations of native and ferrous ion-exposed Rituximab and Trastuzumab to proteins in total lysate of *Bacillus anthracis* was compared (Figure 4B). These analyses revealed that, although both Abs acquire polyreactivity towards the bacterial antigen, Rituximab exhibited a higher capacity to gain novel antigen binding reactivities after exposure to redox-active metal ions as compared to Trastuzumab.

**Figure 5:**
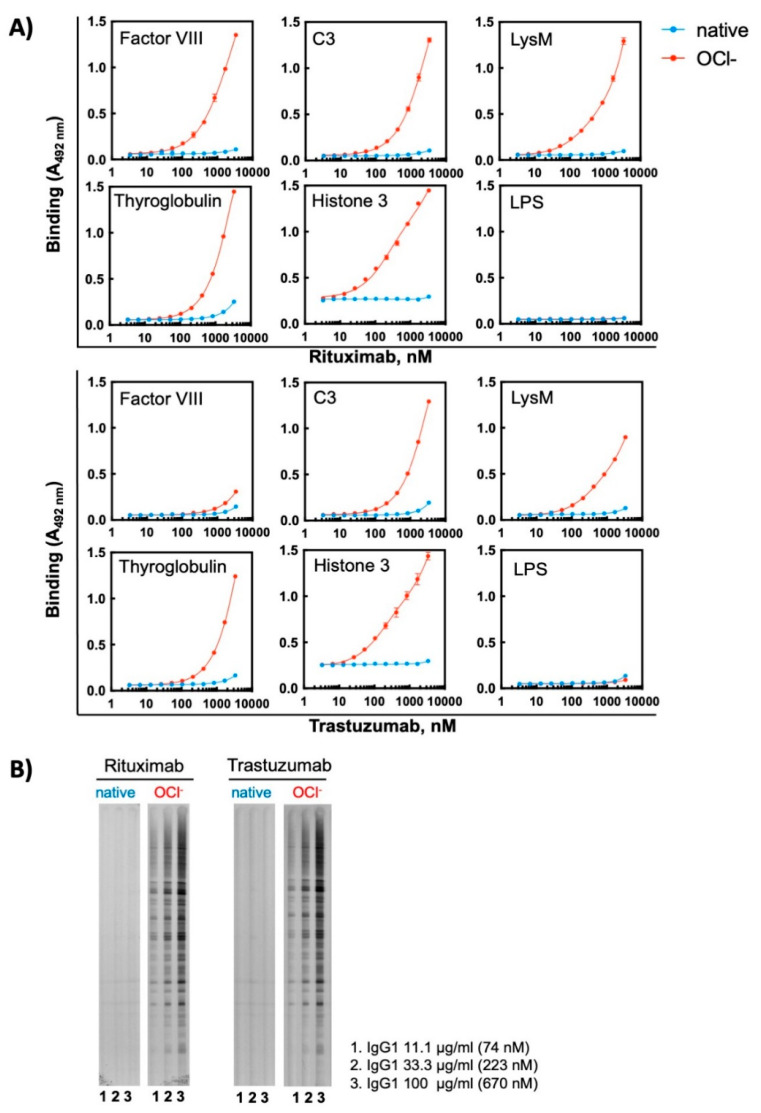
Treatment of therapeutic Abs—Rituximab and Trastuzumab—with hypochlorite ions results in induction of antigen-binding polyreactivity. (**A**) ELISA assay was used to evaluate the reactivity of native and OCl^−^ ion-exposed Rituximab or Trastuzumab. The Abs at 1 mg/mL (6.7 μM) were treated with 200 μM final concentration of NaOCl. After native and hypochlorite ion-treated Abs were serially diluted 3350—3.27 nM and incubated with the indicated proteins and LPS from *E. coli* immobilized on ELISA plates. Each symbol represents the averaged binding intensity obtained from triplicate samples ± SD. (**B**) Immunoblot analyses of reactivity of native and hypochlorite ion-exposed Rituximab and Trastuzumab to antigens from total lysate from *Bacillus anthracis*. Abs were treated at 1 mg/mL (6.7 μM) with 200 μM NaOCl. Next native and treated Abs were diluted to 100, 33 and 11 μg/mL and incubated with the immobilized antigens using *Immunetics* miniblot system.

Ferrous ions can establish metal-coordinative interactions with certain amino acid residues in proteins. These interactions may cause changes in the protein conformation. To dissociate the metal coordination effects from pro-oxidative effect of Fe^2+^ ions, we tested the capacity of unrelated oxidizing substance—hypochlorite ions (OCl^−^)—to induce polyreactivity of monoclonal Abs. Hypochlorite anions represent biologically relevant oxidizing substance produced by enzyme myeloperoxidase in activated neutrophils [24]. Our data showed that exposure of Rituximab and Trastuzumab to sodium hypochlorite resulted in a considerable induction of polyreactivity, as determined by ELISA and Western blot analyses (Figure 5). In similar way to the effect of metal ions, the hypochlorite exposure altered the reactivity of Abs to protein antigens but not to LPS (Figure 5A). As observed for Fe^2+^ ions, Rituximab showed slightly higher antigen reactivity after exposure to hypochlorite by ELISA, for example compare the reactivity of the treated Abs to FVIII and LysM obtained by ELISA (Figure 5A). It should be noted however that this difference was less discernible by immunoblot data (Figure 5B).

Taken together the obtained results suggest that exposure of sensitive Abs to pro-oxidative substances with different chemical nature result in profound functional changes, i.e., gain of binding polyreactivity to unrelated proteins.

### 3.5. Molecular Composition of Monoclonal Abs after Exposure to Pro-Oxidative Substances

**Figure 6:**
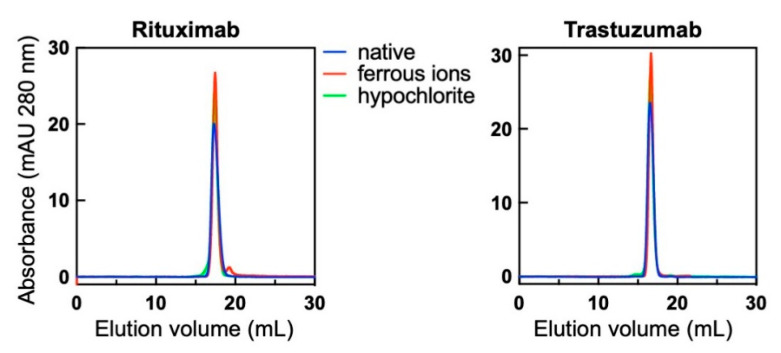
Molecular composition of native Abs and those treated with redox agents therapeutic. Rituximab and Trastuzumab at 1 mg/mL were exposed to 500 μM final concentration of FeSO_4_ or 200 μM NaOCl. Native and treated Abs were injected through Superose 6 size-exclusion profiles and their profiles recorded at 280 nm. Blue lines indicate the profile of native Abs, the red lines of Fe^2+^ ion-treated and green lines OCl^−^ ion-exposed Abs. Of note, Rituximab showed slight retention to the column evident by the larger elution volume of native Ab as compared to Trastuzumab.

Exposure of Abs to pro-oxidative substances can produce protein aggregation, which may have considerable effect on their functional characteristics including antigen-binding behaviour. To assess whether the functional effects observed after treatment of Abs to ferrous ions or hypochlorite are due to aggregation of immunoglobulin molecules, we investigated the molecular composition of the studied Abs by using size-exclusion chromatography. The treatment of Rituximab and Trastuzumab with ferrous ions or hypochlorite at concentrations used for triggering polyreactivity did not result in significant changes in the elution profiles of the Abs (Figure 6). Both Abs retained the elution profiles of their corresponding native monomeric forms. This result indicates that exposure to pro-oxidative substance did not result in formation of high-molecular weight species of Abs and that the observed changes in reactivity are not a consequence of multivalent antigen binding by IgG aggregates.

### 3.6. Reactivity of Therapeutic Abs to Cognate Antigens after Exposure to Pro-Oxidative Substances

**Figure 7:**
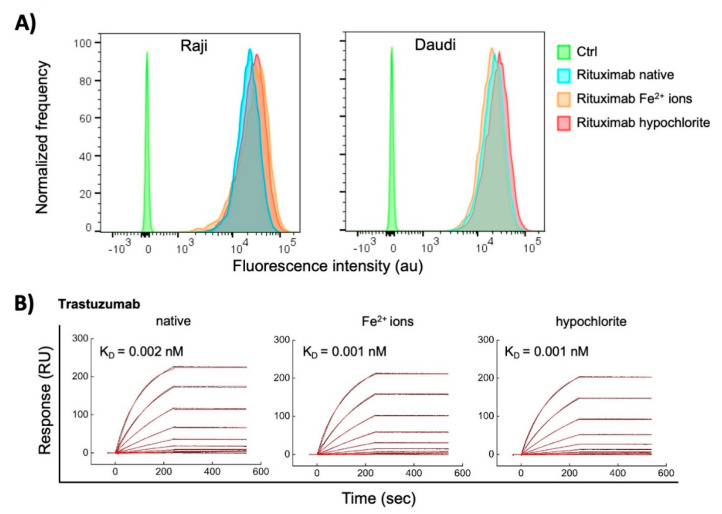
Interaction of native Abs and those treated with redox agents therapeutic with their cognate antigens. (**A**) Flow cytometry analyses of binding of Rituximab to human B cell lymphoma cell lines, expressing CD20. Rituximab at 1 mg/mL was exposed to a 500 μM final concentration of FeSO_4_ or 200 μM NaClO. Native and treated Rituximab at final concentration of 10 μg/mL was incubated with Raji and Daudi cell lines. The histograms depict the normalized frequency of fluorescence intensity indicating the binding of the Ab to the cell. The control (Ctrl) represents the binding of the secondary anti-human IgG to the cells. (**B**) Surface plasmon resonance evaluation of interactions of Trastuzumab. Real-time interaction profiles of native-, ferrous ion- or hypochlorite-treated Trastuzumab to Her2 immobilized on the sensor chip. Trastuzumab at 1 mg/mL was exposed to 500 μM final concentration of FeSO_4_ or 200 μM NaClO. The binding profiles of generated after injection of serial dilutions (10–0.039 nM) of native and treated Trastuzumab are presented. The black curves indicate the experimentally observed binding response, the red curve depict the kinetic fit, using global Langmuir analyses. The values of equilibrium dissociation constant are shown within each graph. All interaction analyses were performed at 25 °C.

The observed dramatic effects of ferrous ions and hypochlorite on the reactivity of some therapeutic Abs elicited the question of how these pro-oxidative substances would influence the binding of Abs to their cognate antigens. To address this matter, we compared the reactivity of Rituximab and Trastuzumab to their respective targets before and after exposure to pro-oxidative substances. Rituximab is Ab-specific for human CD20; the latter is expressed on the membrane of normal and malignant B cells. Trastuzumab is specific for Her2—a cell growth factor receptor overexpressed on some breast carcinoma cells. By using flow cytometry, we compared the reactivity of native and treated with Fe^2+^ ions or hypochlorite Rituximab with CD20 expressed on two different human B cell lymphoma lines Raji and Daudi. The obtained result showed that the recognition of the cognate antigen of Rituximab is not compromised after contact with pro-oxidative substances at concentration used for induction of polyreactivity (Figure 7A). Furthermore, we applied surface plasmon resonance-based approach for assessment the binding kinetics of native and treated with pro-oxidative substances Trastuzumab to covalently immobilized on sensor chip human Her2. The real time interaction profiles and kinetics analyses revealed that the exposure of Trastuzumab to Fe^2+^ ions or hypochlorite did not significantly change the binding to its cognate antigen (Figure 7B). This result corroborates the data obtained by flow cytometry for Rituximab.

Collectively these results demonstrate that the induction of polyreactivity by pro-oxidative agents does not compromise the reactivity of the Abs to their target antigens.

## 4. Discussion

In the present study, we demonstrated that a significant number of the clinical-stage therapeutic Abs experience marked functional changes after exposure to ferrous ions. Thus, the contact with the redox active metal ions resulted in induction of antigen-binding polyreactivity of the sensitive Ab molecules. Correlation analyses revealed that Abs susceptible to the functional changes upon exposure to the metal ions have a higher number of somatic mutations and use with a higher frequency V_L_ domains encoded by the Vκ1 gene family. In contrast, sensitivity to Fe^2+^ ions was significantly less prevalent among Abs that use the Vκ3 gene family for encoding their V_L_ domains. By using two prototypic therapeutic Abs presenting with high sensitivity to ferrous ions in the initial screen of reactivity—Rituximab and Trastuzumab—we observed that, regardless of acquisition of antigen-binding polyreactivity directed to unrelated antigens, exposure to the metal ions did not deteriorate the binding of these Abs to their cognate antigens. In this study we also found that the two prototypic therapeutic Abs showing susceptibility to the pro-oxidative metal ions acquired polyreactivity upon exposure to an unrelated redox-active compound—hypochlorite. Importantly, the induction of polyreactivity of the monoclonal Abs was not associated with aggregation of the IgG1 molecule, as evaluated by size-exclusion chromatography.

In previous studies, it was observed that the exposure of human pooled IgG purified from healthy individuals or a mouse monoclonal IgG (clone Z2) to ferrous ions results in appearance of novel antigen binding reactivities and polyreactivity [16]. Mechanistic studies using the mouse Ab Z2 revealed that induction of polyreactivity by Fe^2+^ ions is associated with an increase in the structural flexibility and the overall hydrophobicity of antigen-binding site [16]. The augmented structural dynamics of V regions induced by the metal ions might explain ability of the Ab to recognize multiple unrelated proteins. It is noteworthy that, despite the changes in the physicochemical characteristics of antigen-binding site, the binding affinity of Z2 to its cognate antigen was not significantly affected after induction of polyreactivity by Fe^2+^ ions [16]. These data corroborate the results obtained in the present study, where we did not detect considerable influence of ferrous ions or hypochlorite on the binding affinity of Rituximab and Trastuzumab to their principal targets (CD20 and Her2, respectively). The experiments with Z2 implied that the retention of the same value of the binding affinity is due to compensatory changes in the binding kinetics during the interaction process. Indeed, as compared to the native Z2, the ferrous ion-exposed Ab recognized its target antigen with a diminished association rate, which has a detrimental effect on the overall affinity. However, these unfavourable changes were reciprocally compensated by the concomitant decrease in the dissociation rate constant, signifying a higher stability of the complex with the antigen [16].

Analogous to effects of ferrous ions, treatment of some IgG molecules with other redox-active substances, including some of in vivo relevance such as heme, hypochlorite, and different ROS, have been shown to induce polyreactivity of human and mouse Abs [15,17,18,20,21,25,26,27], including of a panel of nine therapeutic Abs [28]. Notably, the pathway for achievement of polyreactivity of Abs after contact with iron-containing tetrapyrrole compound, heme, can differ from the one typical for ferrous ions. Thus, our studies implied that heme directly interacts with the antigen-binding site of Abs and by virtue of its high intrinsic reactivity heme can serve as an interfacial cofactor molecule endowing Abs with capacity to bind to multiple antigens [18,29]. The studies with model Ab Z2 indicated that, contrary to the effect of ferrous ions, heme binding rigidifies the binding site of the Ab [18]. Nevertheless, prior studies also suggested that redox activity of the central iron ion in heme is indispensable for its ability to induce polyreactivity of Abs [18,29]. The particularities in the mechanisms of heme and ferrous ions could explain the finding from the present study that, despite the presence of a significant correlation (*p* < 0.0001) between the effects on two substances, this correlation was not very strong (ρ = 0.38) and many Abs demonstrated predominant sensitivity to one of these redox active substances. Moreover, a previous investigation from our group demonstrated that heme-mediated polyreactivity of clinical-stage Abs from the same repertoire used in the current investigation significantly correlated with sequence features in CDR H3 and CD H2 of the heavy chain [23]. These regions in the V_H_ domain are characterized by the highest degree of sequence heterogeneity among Abs and they play the central role in recognition of protein antigens. In contrast the Fe^2+^ ions-mediated polyreactivity was mainly associated with sequence traits in V_L_ domains and more specifically in the framework regions. No significant correlations with sequence features of CDR H3 were found. Another apparent difference between Abs gaining polyreactivity after exposure to heme and to ferrous ions is that sensitivity to ferrous ions positively correlates with the mutation loads in the V_H_ and V_L_ domains. Such a correlation was not observed in the case of therapeutic Abs acquiring polyreactivity upon heme exposure [23].

Vulnerability to oxidation is an important developability hallmark of therapeutic Abs [3,14]. Indeed, the oxidation of the immunoglobulin molecule can cause decrease in thermodynamic stability, increase in aggregation, loss of antigen specificity, increase in immunogenicity, etc. Due to these important issues, numerous studies have been dedicated to elucidation of the effect of forced oxidation of therapeutic Ab or on the development of analytical tools for detection of oxidative modifications in the immunoglobulins [14,30,31,32,33]. These studies revealed that the most susceptible amino acid residues to oxidation in Ab molecules are methionine and tryptophane (especially when exposed on the protein surface) [3,32,34,35,36,37,38]. The oxidative modification of immunoglobulins can occur at different stages of their development—expression, purification, storage, or after administration in patients. Thus, for example Abs can be exposed to redox-active metal ions (including iron ions) due to leakage from metal bioreactors [33]. Investigations using forced oxidation of therapeutic Abs demonstrated that oxidation can cause in some cases loss in antigen binding affinity, whereas in other cases the oxidation did not impact the antigen binding [32,34,39,40]. Oxidative modifications were also shown to impact the Fc-dependent interactions of IgG as for example binding to protein A, and interaction with FcRn, [39,41,42,43,44], thus compromising the half-life and the effector functions of therapeutic Abs [45,46].

In the present study, we observed that contact with pro-oxidative substances can affect some therapeutic Abs in an alternative manner—it induces de novo appearance of antigen-binding polyreactivity. The polyreactivity itself is considered as one of the serious liabilities for therapeutic Abs [22]. Indiscriminative binding to multiple targets can significantly decrease the circulatory half-life of the IgG molecule. In addition, the binding promiscuity of Ab can induce some unwanted secondary reactions. In contrast to other developability issues, the redox agents-induced polyreactivity is not present in the native form of antibodies but it is triggered post-translationally. It is noteworthy that some of the agents able to induce polyreactivity of Abs, e.g., hypochlorite and heme, are present in vivo and their extracellular concentration can be augmented in case of inflammation, tissue damage or hemolysis [24,47]. Indeed, a previous study revealed that induction of local inflammation in mice by injection of complete Freund’s adjuvant cause augmentation of antigen-binding reactivity to unrelated antigens of Abs circulating through the site of inflammation [48]. In this respect, it would be of importance to understand whether the administration of redox-vulnerable therapeutic Abs in patients with conditions that result in release of substances triggering polyreactivity can influence their pharmacokinetics and the therapeutic efficacy. Since the widely used therapeutic Abs—Trastuzumab and Rituximab—are very sensitive to the effect of ferrous ions, it is worth investigating whether lack of efficacy or presence of side effects in some patients can be associated with induction of polyreactivity due to exposure to pro-oxidative conditions in vivo.

Although our study did not provide a mechanistic explanation of how ferrous ions or hypochlorite trigger polyreactivity of certain Abs, the obtained data suggest that Abs with some sequence characteristics are significantly more resistant to pro-oxidative compounds. This finding can be of use for selection of appropriate molecules with good developability profiles at early stages of Ab development programs. Thus, our data show that monoclonal Abs with V_L_ domains encoded by Vκ3 gene family are much more resistant to the effect of the pro-oxidative agent.

In conclusion, our data demonstrate that a substantial fraction of clinical-stage Abs acquire polyreactivity upon exposure to redox-active metal ions or an in vivo-relevant oxidant. By applying statistical analyses, we underlined the sequence features in V domain responsible of susceptibility of Abs to the pro-oxidative substance. The obtained result can contribute for improvement of developability profiles of Ab therapeutics.

## Data Availability

The datasets generated for this study are available on request to the corresponding author.
